# Lung Transcriptomics during Protective Ventilatory Support in Sepsis-Induced Acute Lung Injury

**DOI:** 10.1371/journal.pone.0132296

**Published:** 2015-07-06

**Authors:** Marialbert Acosta-Herrera, Fabian Lorenzo-Diaz, Maria Pino-Yanes, Almudena Corrales, Francisco Valladares, Tilman E. Klassert, Basilio Valladares, Hortense Slevogt, Shwu-Fan Ma, Jesus Villar, Carlos Flores

**Affiliations:** 1 CIBER de Enfermedades Respiratorias, Instituto de Salud Carlos III, Madrid, Spain; 2 Research Unit, Hospital Universitario N.S. de Candelaria, Santa Cruz de Tenerife, Spain; 3 Research Unit, Hospital Universitario Dr. Negrin, Las Palmas de Gran Canaria, Spain; 4 Instituto Universitario de Enfermedades Tropicales y Salud Pública de Canarias, Universidad de La Laguna, Santa Cruz de Tenerife, Spain; 5 Department of Medicine, University of California San Francisco, San Francisco, California, United States of America; 6 Department of Anatomy, Pathology and Histology, University of La Laguna, Santa Cruz de Tenerife, Spain; 7 Septomics Research Centre, Jena University Hospital, Jena, Germany; 8 Section of Pulmonary and Critical Care Medicine, University of Chicago, Chicago, Illinois, United States of America; 9 Keenan Research Center for Biomedical Science at the Li KaShing Knowledge Institute, St. Michael´s Hospital, Toronto, Canada; University of Colorado Denver, UNITED STATES

## Abstract

Acute lung injury (ALI) is a severe inflammatory process of the lung. The only proven life-saving support is mechanical ventilation (MV) using low tidal volumes (LVT) plus moderate to high levels of positive end-expiratory pressure (PEEP). However, it is currently unknown how they exert the protective effects. To identify the molecular mechanisms modulated by protective MV, this study reports transcriptomic analyses based on microarray and microRNA sequencing in lung tissues from a clinically relevant animal model of sepsis-induced ALI. Sepsis was induced by cecal ligation and puncture (CLP) in male Sprague-Dawley rats. At 24 hours post-CLP, septic animals were randomized to three ventilatory strategies: spontaneous breathing, LVT (6 ml/kg) plus 10 cmH_2_O PEEP and high tidal volume (HVT, 20 ml/kg) plus 2 cmH_2_O PEEP. Healthy, non-septic, non-ventilated animals served as controls. After 4 hours of ventilation, lung samples were obtained for histological examination and gene expression analysis using microarray and microRNA sequencing. Validations were assessed using parallel analyses on existing publicly available genome-wide association study findings and transcriptomic human data. The catalogue of deregulated processes differed among experimental groups. The ‘response to microorganisms’ was the most prominent biological process in septic, non-ventilated and in HVT animals. Unexpectedly, the ‘neuron projection morphogenesis’ process was one of the most significantly deregulated in LVT. Further support for the key role of the latter process was obtained by microRNA studies, as four species targeting many of its genes (Mir-27a, Mir-103, Mir-17-5p and Mir-130a) were found deregulated. Additional analyses revealed '*VEGF* signaling' as a central underlying response mechanism to all the septic groups (spontaneously breathing or mechanically ventilated). Based on this data, we conclude that a co-deregulation of '*VEGF* signaling' along with 'neuron projection morphogenesis', which have been never anticipated in ALI pathogenesis, promotes lung-protective effects of LVT with high levels of PEEP.

## Introduction

Sepsis is one of the leading causes of patient admission to the intensive care units (ICUs). It occurs when the initial host response to an infection becomes amplified and deregulated. Coagulation abnormalities, profound hypotension, and multiorgan system dysfunction are the main features of this condition [1], which constitutes the major risk factor for developing acute lung injury (ALI) [2]. Recently, consensus criteria have been reached so that this clinical condition is now referred to as acute respiratory distress syndrome (ARDS) [3]. For simplicity, this study will refer to this condition using the term ALI, irrespective of the severity of the injury. ALI is characterized by intense lung inflammation, increased vascular permeability with diffuse bilateral pulmonary infiltrates, alveolar flooding with a protein-rich edema, severe hypoxemia and reduced lung compliance [4]. The only life-saving lung-directed procedure for these patients is mechanical ventilation (MV) plus moderate to high levels of positive end-expiratory pressure (PEEP). However, it associates with severe complications in up to 70% of patients [4], and leads to fatal outcome in nearly 40% of cases. Previous studies, developed in animal models or cell lines, have focused their attention in elucidating the physiologic and molecular mechanisms underlying the damage that occurs in the lungs after the application of an injurious MV strategy, i.e. with high tidal volume, frequently combined with a lipopolysaccharide (LPS) challenge to resemble a septic status [5]. These studies have supported that the main biological processes underlying lung damage involves immunity, inflammation, apoptosis, activity of pro-inflammatory cytokines, chemotaxis, cell proliferation and coagulation [6, 7–11].

Protective MV using low tidal volumes (LVT) plus PEEP remains the standard management procedure for patients developing ALI. Although the mechanism by which they exert the protective effects is still unknown, it remains the only proven method that reduces mortality [12]. The aim of this study is to identify, for the first time, the molecular mechanisms and pathways that preserve the lungs from the injury, using a clinically relevant sepsis animal model under distinct MV strategies.

## Materials and Methods

### Animal preparation and experimental protocol

The experimental protocol was approved by the Animal Care Committee at the Hospital Universitario N.S. de Candelaria, in accordance with the European Commission Directive 2010/63/EU for animal experimentation. This study followed the ARRIVE guidelines for reporting preclinical animal research [13]. Male Sprague-Dawley rats weighting 300–350 g were included. As previously described [14], animals were anesthetized and paralyzed by intermittent bolus administration of intraperitoneal ketamine/xylazine and pancuronium bromide. Initially, they were randomized into two groups: non septic and septic. Sepsis was induced by cecal ligation and puncture (CLP). The experimental sepsis model started with 25% additional sample size, based on a previous publication by our group using the same CLP model [14], to guarantee a sufficient number of surviving animals at the end of experimental conditions. Twenty four hours post-CLP, surviving septic animals (n = 18) had the cecum removed and allocated for 4 hours to spontaneous breathing (SS, n = 6) or to two strategies of MV: low (6 ml/kg) tidal volume plus 10 cm H_2_O of PEEP (SLVT, n = 6), and high (20 ml/kg) tidal volume with 2 cm H_2_O of PEEP (SHVT, n = 6). We acknowledge that the tidal volume utilized for the SHVT group is far from the one utilized in the clinical setting, however, this choice was based on our previous studies supporting that 20 ml/kg was the minimal level of overdistention in healthy rats causing an identifiable injury comparable to that experienced by ALI patients [14,15]. Furthermore, these studies demonstrated that applying high levels of PEEP to a constant large tidal volume exacerbated lung damage. This evidence challenges the assumption held by some authors that, when moderate to high PEEP levels are used, lung damage can be attenuated in settings where ventilator induced lung injury develops [14,16]. This motivated the use of low PEEP in the SHVT group. Non-invasive monitorization was performed to minimize the possibility of triggering an inflammatory response, providing hemodynamic stability and comparable blood gases in invasively monitored animals during the 4 hours experimental period. Peak airway pressures were continuously monitored and oxygen saturation (SpO_2_) was continuously measured using a pulse oxymeter applied to the rat's tongue. SpO_2_ remained ≥90% in all animals during animal instrumentation. Our previous pilot studies with the model did not evidence significant differences among ventilated groups in the values of mean blood pressure measured through a catheter (ED 0.96 mm; Becton Dickinson, Parsippany, NJ) inserted into the right carotid artery of the rats. Under these conditions, all animals maintained a mean arterial pressure greater than 60 mmHg during the observational or MV period. Therefore, we did not measure continuously arterial blood pressure during the ventilation period in the present study. Healthy, anesthetized, non-ventilated, sham-operated animals (NA, n = 6) served as non-septic controls. Animals were maintained supine on a restraining board inclined 20° from the horizontal. At the end of the 4 hours observation and ventilation period, animals were sacrificed by supplemental pentobarbital (10 mg/kg) and exsanguination after sectioning the abdominal vessels. Then, a midline thoracotomy/laparotomy was performed and the heart and lungs were removed *en bloc*. The lungs were isolated from the heart, washed, snap-frozen in liquid nitrogen and stored for subsequent studies. (See S1 Text for further details)

### Histological examination

In all experimental animals, the right lung was fixed by intratracheal instillation of 3 ml of 10% neutral buffered formalin and then floated in 10% formalin for a week. Lungs were serially sliced from apex to base. Specimens were embedded in paraffin and cut (3 microns thickness) and stained with hematoxylin-eosin. A pathologist examined three random sections of the lung in each animal, with particular emphasis in the alveolar and interstitial damage. Examination was blinded to group identity. Slides were viewed using an Optiphot light microscope (Nikon, Tokyo, Japan) and photographed with a Digital DS-5M camera (Nikon) at x200 magnification.

### Statistical power calculations

Significance Analysis of Microarrays (SAM) [17] was utilized for statistical power calculations for transcriptomic analysis assuming a minimal fold change (FC) between 1.7 and 2.5. We found that selecting as few as three animals *per* group allowed us to achieve a statistical power >80%, to detect a FC of 1.7 for approximately 3000 genes.

### RNA isolation and hybridization

For transcriptomic analysis, total left lung tissue RNA was purified from three surviving animals from each of the four experimental groups. RNA integrity and quantity were assessed using the Bioanalyzer 2100 (Agilent, Palo Alto, CA). RNA integrity was considered suboptimal for one of the non-septic controls (NA), which was excluded from downstream studies. Total RNA was used for cDNA synthesis using First Strand cDNA Synthesis Kit (Roche, Basel, Switzerland) to conduct Real-Time PCR experiments and to produce biotin labeled cRNA. Fragmented cRNA was hybridized to the GeneChip Rat Genome 230 2.0 Array (Affymetrix, Santa Clara, CA). Post-processing quality control analyses flagged one SLVT sample with evidence of higher RNA degradation, which was disregarded from downstream analyses. See S1 Text and S1 Fig for details.

### Differential gene expression analyses with Microarrays

Robust Multi-array Analysis (RMA) algorithm was used for background correction, normalization, and expression levels summarization implemented in the *affy* package [18] for R [19]. The *MulCom* test was used to identify differential gene expression comparing different experimental conditions with a common control condition (NA group) at a False Discovery Rate (FDR) of 0.05 and a minimal FC of 1.7 [20], allowing us to perform a simultaneous comparison among the different septic groups. The complete microarray data set is accessible in the ArrayExpress database (www.ebi.ac.uk/arrayexpress) under accession number E-MEXP-12345.

To validate the probe intensities obtained in the microarray experiments, eight up or down regulated genes were randomly selected for real-time PCR (qPCR) reactions (*S100a9*, *Reg3b*, *Gadd45a*, *Aqp5*, *Irak2*, *Wnt5b*, *Gata6*, and *Serpina3n*). This method is commonly used for validation purposes of probe intensities obtained with the microarrays [21]. Briefly, TaqMan Gene Expression Assays (Life Technologies, Carlsbad, CA) were carried out in triplicate and qPCR efficiencies were calculated for each gene on a 7500 Fast Real-Time PCR System (Life Technologies). β-2 microglobulin was used as the housekeeping gene. The correlation between normalized values obtained by qPCR and the normalized intensities from the microarrays were estimated using the Spearman correlation coefficient in R. See S1 Text for further details.

### Identification of biological processes from differential gene expression analyses

In order to identify biological processes underlying the differentially expressed genes according to microarray intensities, functional annotation clustering analyses in the Database for Annotation, Visualization and Integrated Discovery (DAVID) v6.7 was performed [22], using the Rat Genome 230 2.0 as background. The overlapping genes from all the biological processes detected (FDR<0.05) were then utilized in EnrichNet [23] to provide a condensed view by modeling the underlying protein network structure. For that, we used a human protein-protein interaction network obtained from STRING [24] and the pathways and processes obtained from Reactome [25]. See S1 Text for details.

After the deregulated biological processes were identified, we accessed publicly available genomic data to evaluate if there were common features from our experimental model and independent observations in human studies. Such validation was assessed with two independent pathway analyses: one in a human transcriptomic study in peripheral blood from septic patients with and without ALI [26], and another in the only available genome-wide association study (GWAS) in ALI published to date [27]. For both, Gene Set Enrichment Analysis (GSEA) [28], which determines whether *a priori* defined set of genes shows statistically significant differences between two biological states, was used to obtain the significance of the enrichment in the two datasets, adjusting for multiple testing based on FDR. See S1 Text for details.

### Inference of deregulated microRNA species and biological validation by small RNA sequencing

MicroRNAs (miRNAs) have been proposed as biomarkers for severity in septic patients [29] and in other fibroproliferative diseases [30]. Thus, we wanted to assess if these master regulators could be responsible for the deregulated processes observed in our experimental model. GSEA was also used to infer the abundance of 3'-UTR miRNA binding motifs among the differentially expressed gene lists obtained from microarray studies.

Small RNA sequencing (sRNA-seq) was conducted to validate the miRNAs detected by the bioinformatic GSEA predictions in the same samples employed for microarray studies. Assuming an extensive overlap among the biological processes deregulated among experimental groups, we sequenced the samples from NA and SHVT as their comparison gave the largest differences in gene expression levels. After enrichment of the small RNA fraction, sequencing was conducted in the resulting libraries using the Ion Torrent Personal Genome Machine (Life Technologies). Post-sequencing analyses, including the analysis of differential expression using ANOVA, were assessed on the Torrent Suite v4.0.2 (Life Technologies), Partek Flow and Partek Genomic Suite v6.6 (Partek Inc., St. Louis, MO). As a final step, to deduce the biological processes being targeted by these miRNAs, MsigDB was then used to retrieve the target genes and DAVID was employed to perform an enrichment analysis. See S1 Text for details.

## Results

### Lung injury under different MV strategies

**Fig 1:**
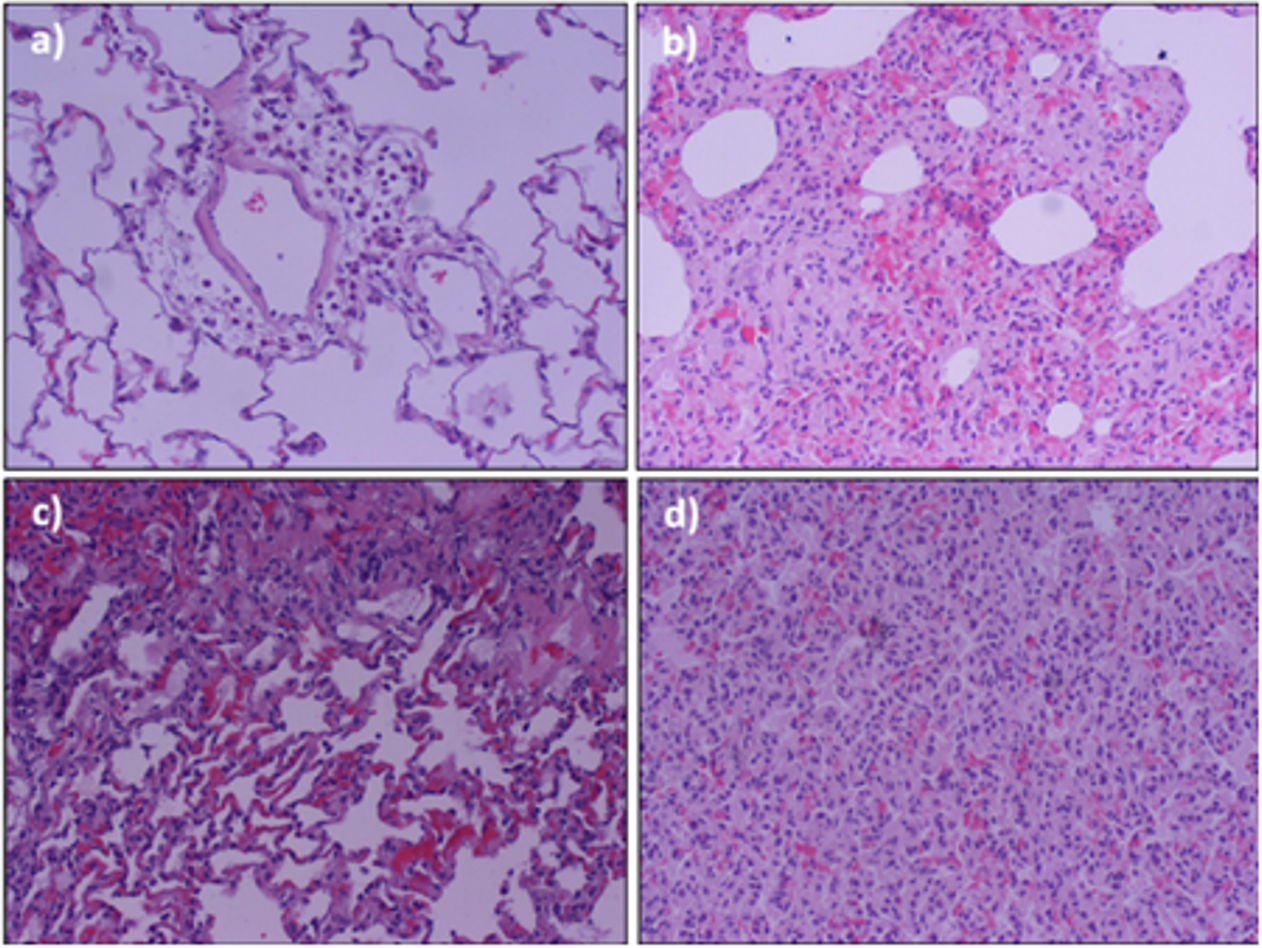
Microscopy images stained with hematoxylin-eosin showing representative lung histopathological features under different strategies of MV: a) non-ventilated non-septic controls (NA); b) Septic, anesthetized, spontaneous breathing after 4 hours (SS); c) septic after 4 hours of MV at LVT + 10 cm H_2_O PEEP (SLVT); and d) septic after 4 hours of MV at HVT + 2 cm H_2_O PEEP (SHVT).

The degree of lung injury differed among septic animals, including atelectasis, pulmonary edema, and acute inflammatory infiltrates. In ventilated animals, gas exchange was impaired, showing a ratio of partial pressure arterial oxygen and fraction of inspired oxygen (PaO_2_/FiO_2_) <250 mmHg at the end of 4 hours of MV, indicative of ALI. The SLVT group had less lung injury than the SS animals, suggestive of the additive effects of mechanical and systemic damage. The SHVT animals had the most severe injury in terms of pulmonary infiltrates, atelectasis and hemorrhage (Fig 1). These results overlap extensively with those from previous studies reported by our group using this experimental model [14,16], and evidence the protective mechanical effect of LVT on severely injured lungs.

### Signaling by VEGF underlies lung gene expression differences among experimental groups

**Fig 2:**
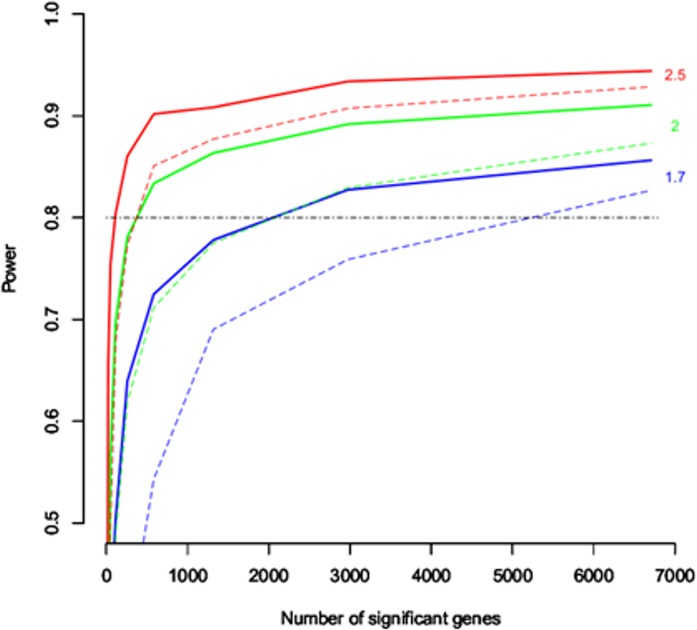
Plot illustrating the statistical power calculations for the study assuming four (two animals per group; dashed lines) and five samples (groups with two and three animals; solid lines), and a mean FC of 1.7, 2 and 2.5.

**Fig 3:**
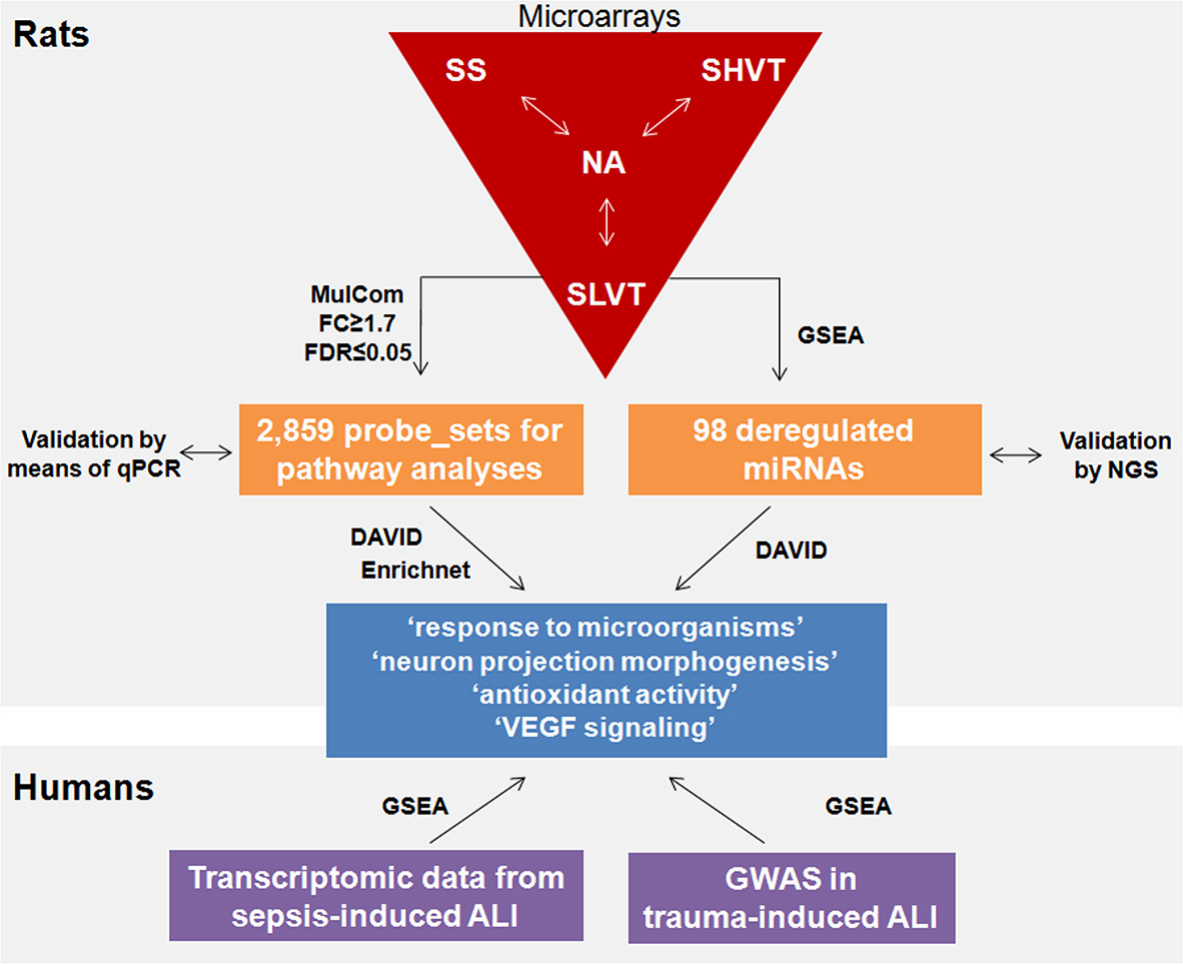
Flow diagram of the experimental design summarizing the transcriptomic analyses, including the validation steps, main methods and results.

**Fig 4:**
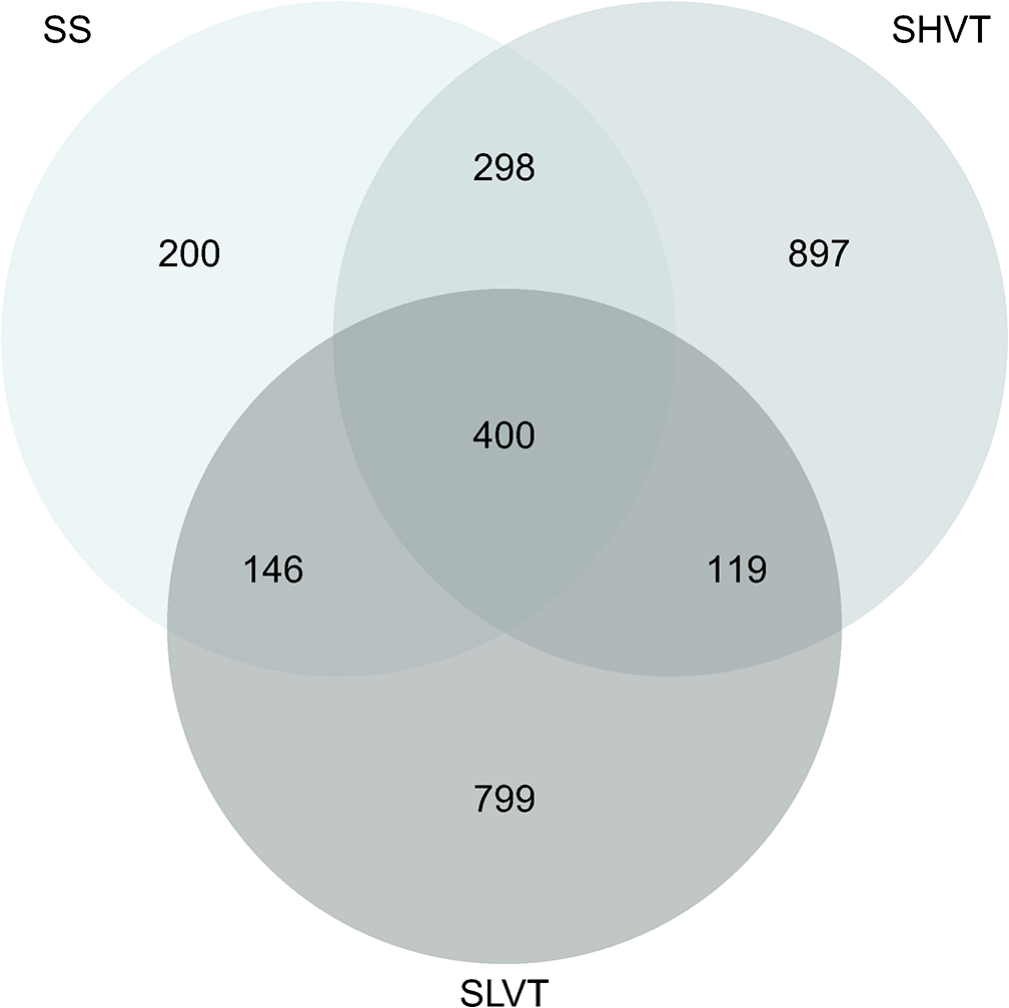
Venn diagram illustrating the distribution of statistically significant changes in gene expression and their overlap among experimental groups. SS: Sepsis spontaneous breathing, SLVT: septic with low tidal volume MV, SHVT: septic with high tidal volume MV. All the comparisons were made against the non-septic controls (NA).

**Fig 5:**
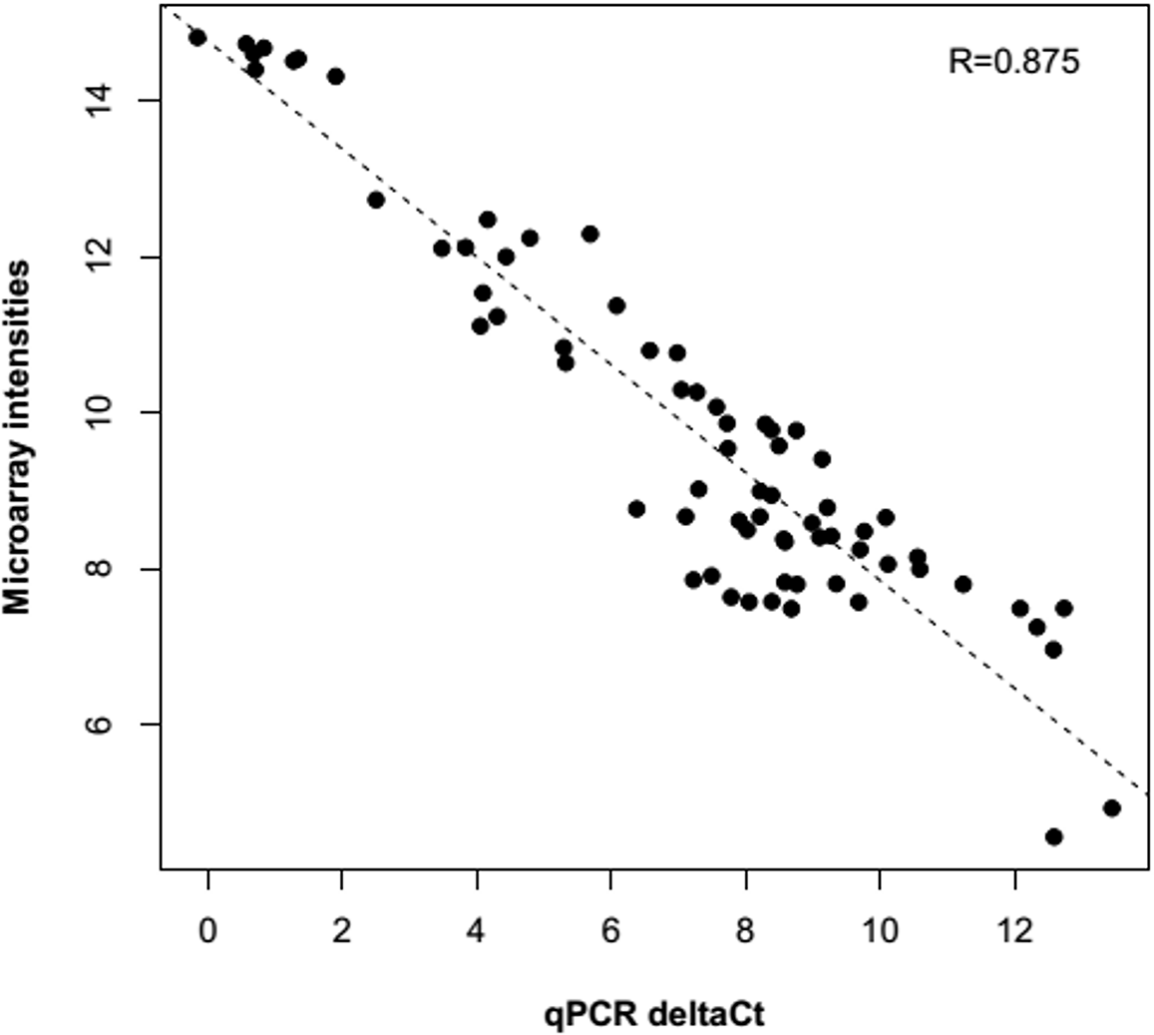
Real time PCR validation of microarray results for eight deregulated genes. The plot represents the correlation among the normalized microarray intensities (y-axis) and cycle threshold values from Real time PCR (x-axis).

After quality control procedures, gene expression analyses were finally performed with a total of ten animals (S1 Fig). Based on power calculations, such reductions in the final sample size still ensured sufficient statistical power (>76%) for downstream transcriptomic analyses (Fig 2). A complete flow diagram showing the main steps of these analyses and the main results of our study are depicted in Fig 3. Differential gene expression revealed 2,859 deregulated probe sets with FDR≤0.05 and FC≥1.7 in the overall experiment with respect to the control (NA): 1,044 in SS group, 1,464 in SLVT group and 1,714 in the SHVT group (S1 Table and Fig 4). A comparison with SAM [17], an alternative and widely used algorithm for differential expression experiments, provided >85% overlap in the deregulated probe sets (S2 Fig). Furthermore, the validity of the microarray results was supported by the high correlation observed for eight deregulated genes between ∆Ct values obtained by qPCR and their normalized microarray intensities (Spearman rank, R = 0.875) (Fig 5).

**Fig 6:**
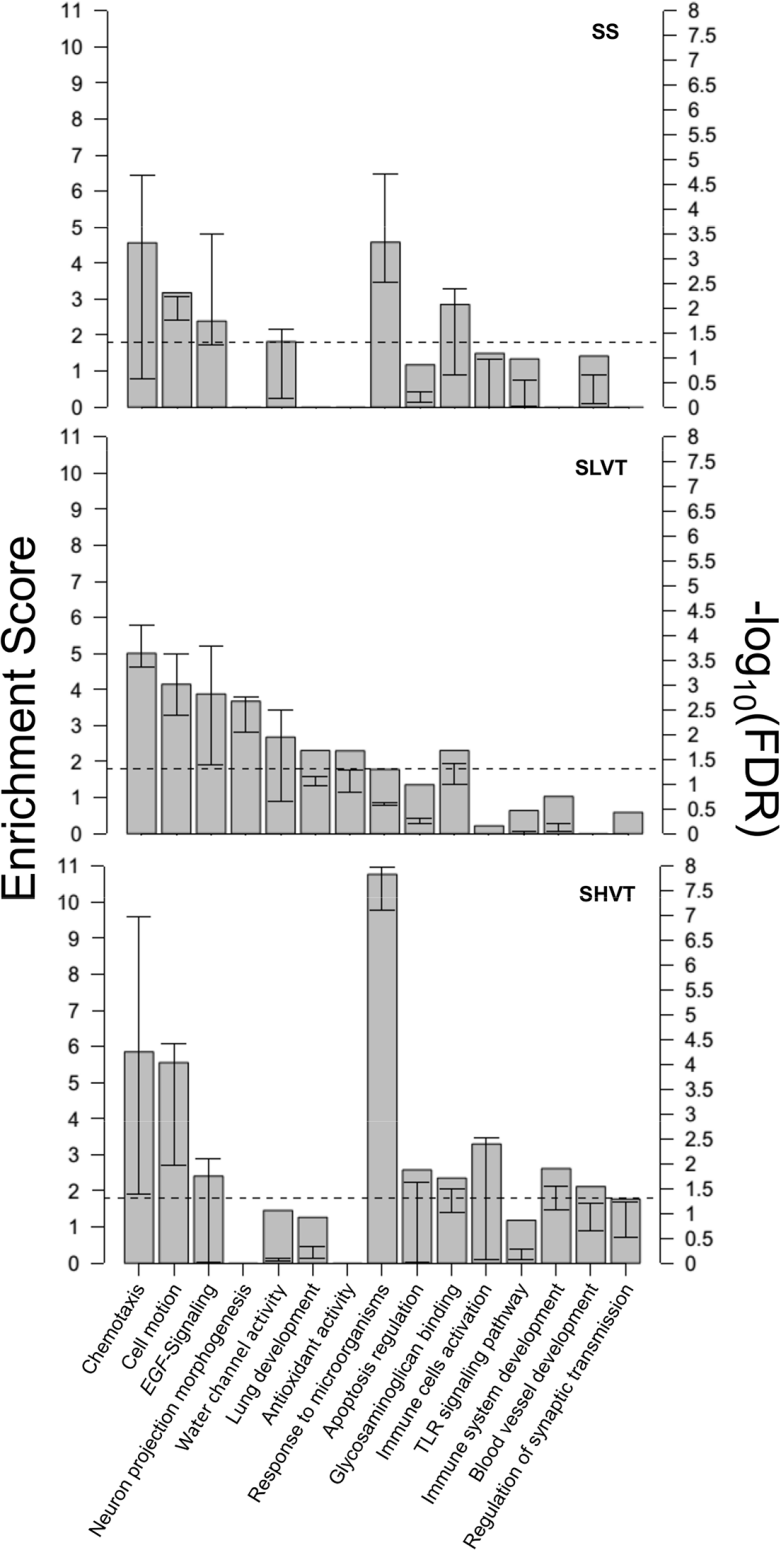
Biological processes associated with significantly deregulated genes compared to the non-septic controls in each experimental group (*x*-axis). The enrichment score (left *y*-axis), a log transformation of the geometric mean of all the enrichment *p*-value, indicates the processes that play major roles in each group. The right *y*-axis reflects a transformation of the FDR to globally correct the enrichment *p*-value for the pathways included within each biological process. Whiskers represent their lower and upper FDR values. The horizontal discontinuous line represents the transformed value for a FDR = 0.05.

Using the list of deregulated genes *per* group, we then assessed their contribution on biological processes, as this could be more informative to reveal the mechanisms that are being activated or repressed during ALI than focusing on particular genes. As expected, several biological processes were found deregulated with an extensive overlap among the three groups. However, the levels of deregulation *per* group were different in most cases (Fig 6). Given that the experimental model involved systemic inflammation, we anticipated that the ‘response to microorganisms’ would be one of the main deregulated processes among the septic groups. However, although it was detected as the leading process in SHVT and SS (FDR<1x10^-3^ in both comparisons), it was not significantly enriched in SLVT (FDR = 0.245). These results reflect again the additive effects of mechanical and systemic damage in the SHVT. Noteworthy, a few unanticipated processes such as the ‘neuron projection morphogenesis’ and the ‘antioxidant activity’ were evidenced in the SLVT group (FDR = 0.002 and FDR = 0.05, respectively).

In light of these results, we then aimed to expose a common underlying mechanism with a pivotal role in the disease pathogenesis, i.e. a mechanism that could explain the observed differences, exacerbating the lung damage, as in SHVT, or protecting the lung from the injury, as in SLVT. For that purpose, a protein-protein interaction network analysis was performed based on the set of deregulated genes shared among experimental groups. This revealed the ‘signaling by vascular endothelial growth factor (*VEGF*)’ as the only mechanism shared across all three conditions with experimental sepsis. Other signaling pathways were also significant in at least one group, but were mainly related to the inflammatory response to pathogens (Table 1). Among these, the evidence supported the role of 'chemokine receptors bind chemokines', 'MYD88 cascade', and the 'toll like receptor 4 (TLR4) cascade'.

**Table 1:** Protein interaction network analysis.

	SS^a^		SLVT^a^		SHVT^a^	
*Annotation (pathway/process)*	XD^b^	FDR^c^	XD^b^	FDR^c^	XD^b^	FDR^c^
Signaling by VEGF	2.38	0.009	2.37	0.040	1.53	0.137
Chemokine receptors bind chemokines	—	—	—	—	1.22	1.73x10^-5^
Prostanoid hormones	—	—	1.55	0.329	—	—
Toll like receptor 4 cascade	1.59	0.001	—	—	1.56	0.001
MYD88 cascade	1.35	0.041	—	—	1.31	0.044
P75NTR signal via NFKB	—	—	—	—	1.97	0.020
Viral DSRNA TLR3 TRIF complex activates RIP1	—	—	—	—	2.14	0.019
Human TAK1 activates NFKB by phosphorylation and activation of IKKS complex	—	—	—	—	1.69	0.026

^a^SS: Sepsis spontaneous breathing; SLTV: septic with low tidal volume mechanical ventilation; SHVT: septic with high tidal volume mechanical ventilation.

^b^Network interconnectivity score indicating the pathways/processes that have functional association with the defined gene set.

^c^Adjusted *p*-value for multiple testing using a False Discovery Rate.

In order to validate the deregulated processes detected in the experimental model, we assessed data from independent published genomic studies of ALI in humans following similar procedures. Strikingly, the results from GSEA in whole blood [26] revealed that signaling by semaphorin and netrin were underrepresented in patients with sepsis-induced ALI. Given that semaphorin and the netrin receptor were both comprised in the 'neuron projection morphogenesis' pathway, this constituted the only biological process overlapping with those from the animal experimental model. Besides, among the association results from the only published GWAS in ALI [27], genes involved in neuron differentiation were also overrepresented among the nominally significant hits, including those encoding molecules implicated in neural and vascular functions (S2 Table), again being the only process overlapping with the results from the microarray studies in the sepsis animal model.

### Altered biological processes and miRNA regulation

To assess if there were particular miRNA species acting as master regulators that could be responsible for the deregulated processes observed in our experimental model, GSEA analysis based on microarray gene expression data was performed. This evidenced 3 and 52 miRNA species underrepresented in SS and SLVT, respectively, compared to NA. In contrast, a total of 43 miRNAs were overrepresented in the SHVT group compared to NA As these were all *in silico* predictions based on the sequences from the 3’-untranslated region (3’UTR) from deregulated genes, we then performed sRNA-seq to identify the most prominent miRNA species out of those identified by GSEA. We have previously noted that genes that were oppositely deregulated in any pairwise comparison were not pervasive among experimental groups (only two probe sets were oppositely deregulated, see S1 Table), thus having a minor contribution to the degree of lung injury. Given this, and that the comparison of SHVT vs. NA yielded the most significant results (the least and the worst damaged animal groups), subsequent sRNA-seq analysis to validate miRNA predictions was performed focusing on these two groups. A heatmap built from nominally significant miRNAs between SHVT and NA detected by sRNA-seq are shown in S3 Fig. When comparing the direct sequencing of the samples with the bioinformatic prediction, 28 miRNA species overlapped, from which Mir-27a, Mir-103, Mir-17-5p, Mir-130a, and Mir-155 were nominally significant although the abundance of the latter was observed to be opposite to the one deduced by GSEA (Table 2). Besides general processes related to gene transcription, a total of 159 genes were targeted by these miRNAs, involving ‘neuron projection morphogenesis’ as the main deregulated biological process. Interestingly, this process was the only one involving all four miRNA species with a consistent abundance among microarray-based predictions and sRNA-seq experiments (Mir-27a, Mir-103, Mir-17-5p and Mir-130a) (FDR<1x10^-4^) (S3 Table). Other deregulated biological processes included ‘blood vessel development’ (Mir-155, Mir-17-5p and Mir-130a) (FDR = 6x10^-4^), 'lung development' (Mir-17-5p and Mir-27a) (FDR = 4x10^-4^), and ‘cell motion’ (Mir-103) (FDR = 8x10^-4^) (S3 Table). A total of 29 genes out of the 159 genes targeted by these miRNAs were deregulated in our microarray results, representing a 4-fold enrichment compared to those expected by chance (Fisher exact test *p* = 3x10^-4^). This reinforces the key role of these biological processes in our experimental model of ALI.

**Table 2:** Overlapping miRNAs among GSEA and sRNA-seq analyses.

miRNA	NES^a^	GSEA (*p*-value)^b^	sRNA-seq (Fold change)	sRNA-seq (*p*-value)^c^
Mir-23a	1.7	2.36x10^-4^	1.36	0.132
Mir-142-5p	1.56	0.005	-1.45	0.321
Mir-181a	1.55	0.001	1.41	0.161
Mir-190	1.54	0.034	1.58	0.055
Mir-27a	1.54	0.001	3.22	**4.3x10** ^**-3**^
Mir-133a	1.54	0.005	-3.3	0.166
Mir-191	1.53	0.046	1.5	0.081
Mir-224	1.51	0.019	1.2	0.36
Mir-103	1.51	0.014	1.58	**0.042**
Mir-107	1.51	0.014	1.84	0.696
Mir-101	1.49	0.007	2.29	0.117
Mir-203	1.46	0.012	1.22	0.332
Mir-155	1.43	0.034	-2.23	**0.019**
Mir-30a-5p	1.43	0.004	1.39	0.28
Mir-199a	1.42	0.04	-1.1	0.511
Mir-18a	1.42	0.045	1.59	0.198
Mir-153	1.41	0.029	7.57	0.342
Mir-128a	1.36	0.034	-1.25	0.559
Mir-194	1.35	0.083	2.3	0.112
Mir-337	1.34	0.061	-3.87	0.196
Mir-22	1.32	0.058	1.14	0.683
Mir-490	1.32	0.132	-2.5	0.064
Mir-139	1.32	0.087	1.28	0.237
Mir-17-5p	1.33	0.023	1.25	**0.046**
Mir-130a	1.31	0.04	2.2	**0.012**
Mir-206	1.3	0.047	2.38	0.162
Mir-144	1.3	0.078	-1.41	0.549
Mir-299-5p	1.29	0.156	1.07	0.95

^a^Enrichment score, reflecting the degree to which a gene set is overrepresented in the SHVT group after 10^4^ permutations.

^b^Nominal *p*-value.

^c^In bold, overlapping and significant miRNA species among analyses.

## Discussion

The current study highlights altered gene expression and deregulation of molecular pathways in the lungs of CLP-induced septic animals when distinct ventilatory strategies are applied, including a protective ventilatory regime for which genomic studies are still lacking. Analysis of the microarray data in lung tissues identified *VEGF* signaling as a key underlying mechanism common to several molecular processes enriched in the experimental model, particularly highlighting ‘neuron projection morphogenesis’ as one of the main deregulated processes under MV with LVT and high levels of PEEP. A number of molecules of this process are involved in neuron differentiation, participating in axon guidance and acting as receptors in neurogenesis [31]. The deregulation of genes encoding these molecules in ALI was further validated in humans by parallel analyses in existing genomic and transcriptomic data from critically ill patients, as well as by additional miRNA analyses by *in silico* predictions and sRNA-seq validations in lung tissues from a subset of animals.

Injured lungs from ALI patients exhibit diffuse alveolar damage that occurs in several phases including an exudative and a proliferative phase, the latter characterized for fibroblastic proliferation. Multiple pathways are involved in the resolution of existing and progressing fibroproliferative diseases [32], which will ultimately lead to different outcomes when they become deregulated. VEGF is activated during wound healing and repair, modulates several aspects of the immune and inflammatory responses [33], stimulates endothelial cell (ECs) migration [34], acts as a chemotactic factor for certain human immune cells [35], and induces transient vascular leakage [36]. These activities place VEGF as a central player regulating barrier dysfunction, microvascular pulmonary ECs [37] and having key roles during acute and resolving lung injury in humans [38]. All these processes were significantly deregulated in our experimental animal model and might be determinants of the paradoxical role of VEGF both during lung damage and repair [39]. VEGF receptors and co-receptors are expressed on ECs that rapidly respond to hypoxia and inflammation and reactivate the angiogenic process to regenerate the damaged tissue. However, chronic inflammation can trigger an excessive accumulation of extracellular matrix components and lead to the formation of a permanent fibrotic scar [40]. We hypothesize that this could be an underlying process in SHVT, where a co-deregulation of VEGF signaling and inflammatory responses due to the 'response to microorganisms' might activate remodeling processes in the lung. Previous findings supports that alveolar overdistention itself, may trigger an immune response similar to that observed during severe infections through TLR4 interaction [41]. Interestingly, the TLR4 cascade was identified in the protein-protein network analysis, and was deregulated in SHVT and SS groups, but not in SLVT. Therefore, the observed inflammatory exacerbation in the experimental model is due to both, the direct response to microorganisms and to the MV-related mechanical damage [42].

On the other hand, the knowledge of the molecular mechanisms leading to lung repair is scarce [43]. Nerves and vessels share comparable patterning and angiogenic molecules, morphogens, and growth factors that have dual roles in vascular and neuronal development [44,45]. These molecules are termed angioneurins [45] and VEGF is classified as one, along with semaphorins, netrins, slits, ephrins and neuropilins [31]. All of these molecules or their receptors were deregulated in the 'neuron projection morphogenesis’ process in the SLVT group, highlighting their potential implication in repairing processes observed in this MV strategy. Furthermore, the participation of these molecules was also validated in available human genomic and transcriptomic data, even when the latter was obtained from whole blood instead of lung tissue and gene expression is not always comparable, increasing the confidence of our results. Interestingly, there are also several evidences in the literature relating hypoxia with angiogenesis induction and axon guidance modulation [31]. Hypoxia is the main regulator of VEGF expression as it is a direct transcriptional target of both hypoxia inducible factor (HIF) 1α and HIF-2α [46]. In pigs and humans, genes encoding these molecules have been involved in adaptation to high altitude, one of the most extreme environments characterized by low concentrations of atmospheric oxygen (hypoxia) [47–49]. Additionally, neuroepithelial bodies (NEBs) are clusters of pulmonary neuroendocrine cells that are highly innervated and may act as complex sensors of hypoxia [50]. NEBs harbor and co-localize with pulmonary progenitor cells [51], capable of epithelial regeneration in different models of lung injury [52].

Indirect evidence from miRNA analyses (both *in silico* and sRNA-seq validation) also bolstered the role of 'neuron projection morphogenesis' as one main biological process involved in ALI development in our experimental model, based on the enrichment of genes targeted by key miRNA species that were significantly deregulated in the microarray studies. In this sense, by virtue of miRNAs known mechanism of action, reducing gene expression by binding to the 3'UTR of their targeted genes, a number of evidenced miRNA species (Mir-27a, Mir-103, Mir-17-5p and Mir-130a) might be involved in turning off the 'neuron projection morphogenesis' process in the SHVT group. This may explain why it was found deregulated exclusively in the SLVT group. Further exploration of the role of these pathways will be needed to confirm its involvement in tissue repair during the setting of ALI. However, in support of these observations, common variants on *VEGF* gene have been previously associated with ALI susceptibility or outcomes in critically ill patients, and the results have been confirmed by independent studies [53], emphasizing its role in the disease status or progression.

There are several limitations of this study. The purpose of the study was to unravel the biological mechanisms occurring in the lungs under physiological conditions in a clinically relevant experimental model. This motivated the use of whole lung tissue, instead of lung structural cells, for transcriptomic analysis. Therefore, the experiments exposed a compendium of the biological processes occurring in all cell types present in the challenged tissues, including structural and infiltrated inflammatory cells, resembling the physiological conditions in the lungs. We recognize that the sample size utilized for transcriptomic analyses was scarce. However, this only affected the detection capacity by lowering the dynamic range of gene expression, so that only moderate to drastic changes could be detected. In fact, our observations in the rodent experimental model were paralleled with evidences inferred from distinct genomic data from humans, supporting our results and increasing their potential clinical value. Besides, in a recent study of miRNA and gene expression in an animal model of ARDS [54], several deregulated miRNAs in that model overlapped with the results obtained from *in silico* predictions and sRNA-seq validation performed here.

The main advantage of this study is that this is the first time the molecular mechanisms of lung injury induced by sepsis after CLP are explored. The CLP is considered as the gold standard model as it closely mimics the pathophysiology of human sepsis and ALI [55,56], including insidious onset, hyperdynamic cardiovascular state, reversible compromised myocardial performance and a high mortality rate [57]. On the other hand, we acknowledge that the tidal volume utilized for the SHVT group is far from the one utilized in the clinical setting. This choice was based on the evidence suggesting that rodents are highly resilient to inflammatory challenges [58]. In agreement, our pilot studies supported that this high tidal volume produced an identifiable injury compared to the one experienced by ALI patients in nondependent areas of the lungs [14,59].

## Conclusions

To the best of our knowledge, this is the first transcriptomic study of ALI to integrate the sepsis state, MV, data from miRNA deregulation, and existing publicly available transcriptomic and GWAS data. These observations support that VEGF and its receptors are just the tip of the iceberg triggering mechanisms involved in tissue repair. This information will eventually translate in useful clinical information for the ventilatory management of critically ill patients.

## Supporting Information

S1 Fig Flow diagram of the experimental design and samples utilized for microarray hybridization in the animal model of sepsis induced by cecum ligation and puncture. Among the septic rats, eighteen underwent mechanical ventilation (MV) using high and low tidal volume. *Note: Among the low tidal volume group, array post-processing quality controls indicated that RNA from one sample might be degraded and, therefore, was discarded from further analyses.
(TIFF)

S2 Fig Venn diagram showing significant overlapping probes among the Significance Analysis of Microarrays (SAM) and the MulCom test at False Discovery Rate = 0.05 and Fold change≥1.7. (TIFF)

S3 Fig Hierarchical clustering of altered miRNA species determined to be nominally significant when comparing SHVT (HighVent) to the NA group (Control). (TIFF)

S1 Table Significant probe sets in the microarray-based differential gene expression analysis comparing experimental sepsis groups with a common control (NA) group. (XLS)

S2 Table Genes involved in the 'neuron differentiation' process (GWAS-based GSEA overrepresentation) and their top significant polymorphism in the discovery phase of the association study. (PDF)

S3 Table Biological processes targeted by significant microRNAs that overlapped between microarray-based predictions and sRNAseq experiments. (XLS)

S1 Text Supplementary methods. (DOC)
