# Correction: Lung Transcriptomics during Protective Ventilatory Support in Sepsis-Induced Acute Lung Injury

**DOI:** 10.1371/journal.pone.0145696

**Published:** 2015-12-17

**Authors:** Marialbert Acosta-Herrera, Fabian Lorenzo-Diaz, Maria Pino-Yanes, Almudena Corrales, Francisco Valladares, Tilman E. Klassert, Basilio Valladares, Hortense Slevogt, Shwu-Fan Ma, Jesus Villar, Carlos Flores

The accession number for the complete microarray data set deposited in ArrayExpress appears incorrectly throughout the article as E-MEXP-12345. The correct accession number should be E-MTAB-2673.
