# Supplementary figures and images for: Lung Transcriptomics during Protective Ventilatory Support in Sepsis-Induced Acute Lung Injury
Source: PLoS One. 2015 Jul 6;10(7):e0132296. doi: 10.1371/journal.pone.0132296 (PMC4492998; doi:10.1371/journal.pone.0132296)

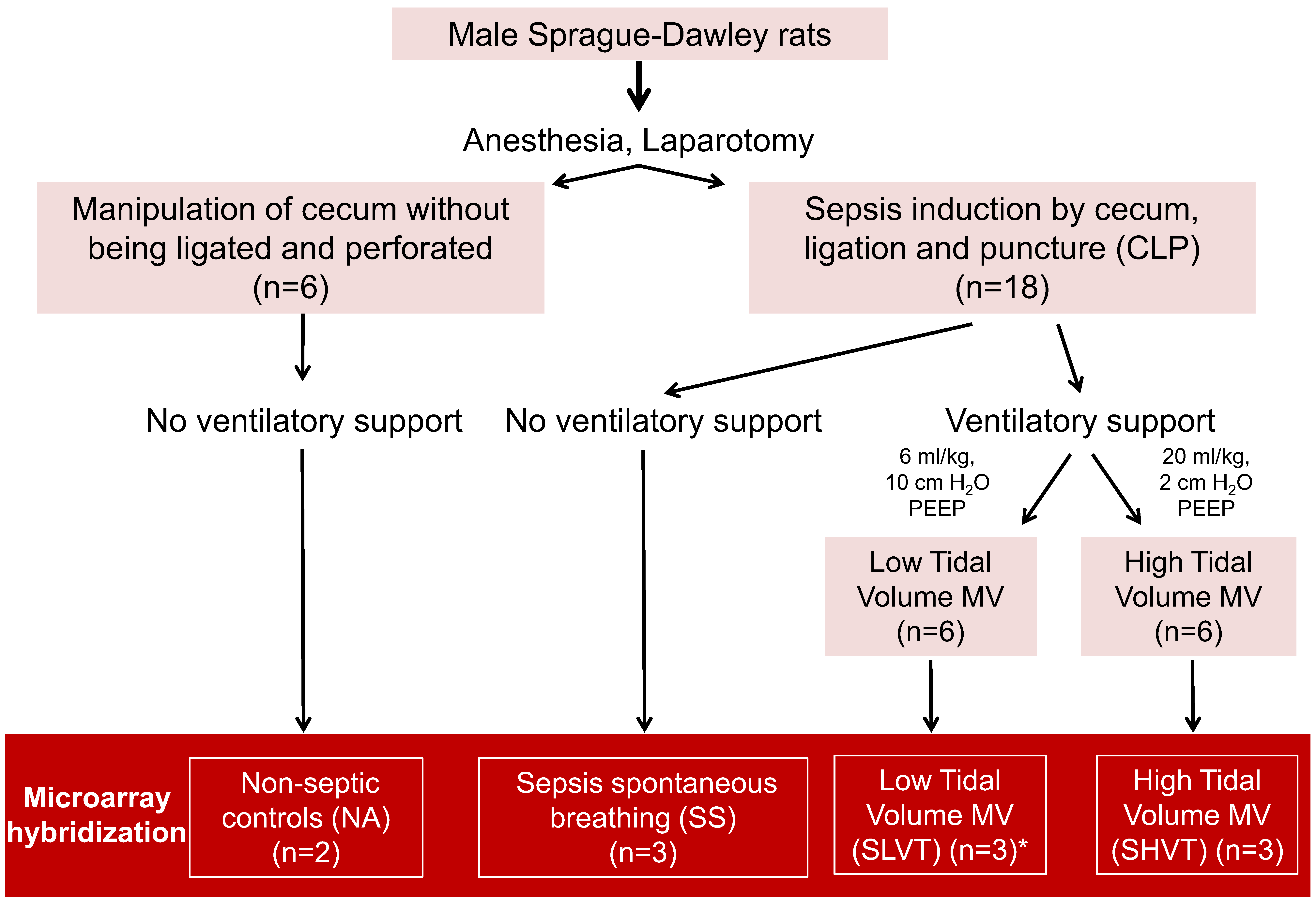

Supplement: S1 Fig — Among the septic rats, eighteen underwent mechanical ventilation (MV) using high and low tidal volume. *Note: Among the low tidal volume group, array post-processing quality controls indicated that RNA from one sample might be degraded and, therefore, was discarded from further analyses. (TIFF) [file pone.0132296.s001.tiff]

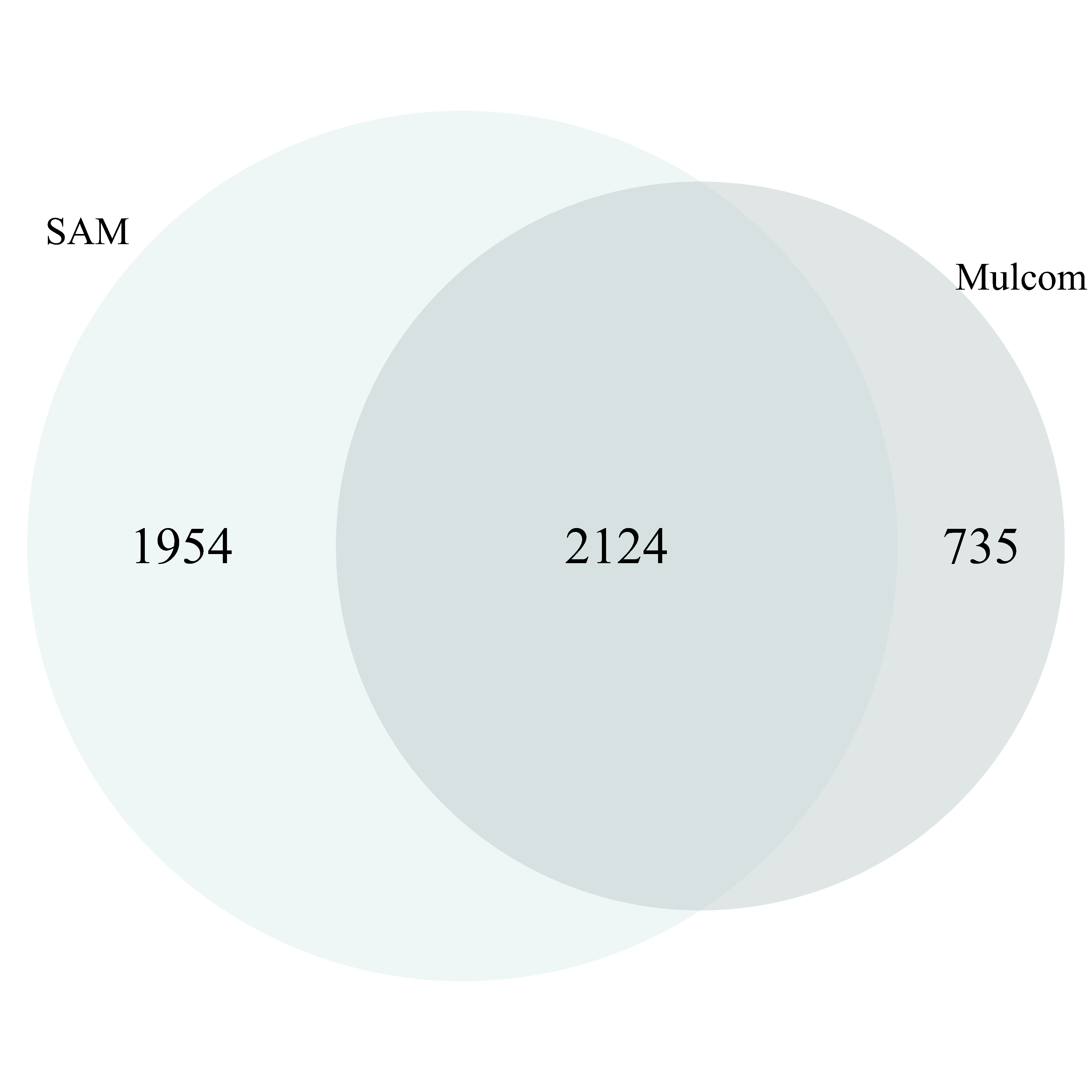

Supplement: S2 Fig — (TIFF) [file pone.0132296.s002.tiff]

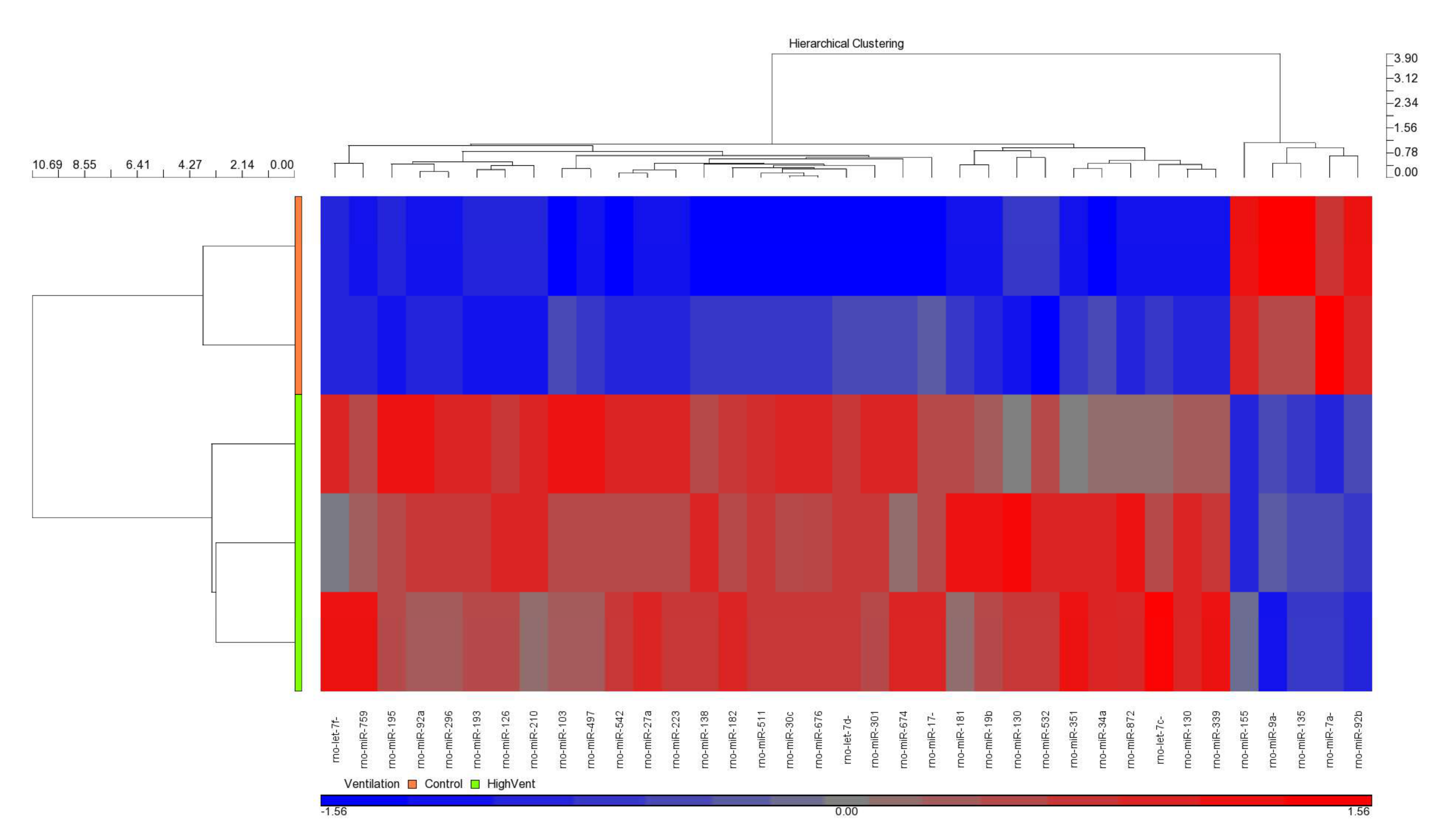

Supplement: S3 Fig — (TIFF) [file pone.0132296.s003.tiff]
