# Supplementary material for: Lung Transcriptomics during Protective Ventilatory Support in Sepsis-Induced Acute Lung Injury
Source: PLoS One. 2015 Jul 6;10(7):e0132296. doi: 10.1371/journal.pone.0132296 (PMC4492998; doi:10.1371/journal.pone.0132296)
Supplement: S2 Table — (PDF) [file pone.0132296.s005.pdf]

Table S2. Genes involved in the 'neuron differentiation' process (GWAS-based GSEA overrepresentation) and their top significant polymorphism in the discovery phase of the association study.

Gene name	Mapped polymorphism	GWAS *p*-value
NRXN3	rs12587614	$1.40x10^{-4}$
ATP2B2	rs7629483	$3.29x10^{-4}$
ROBO1	rs7611200	$3.35x10^{-4}$
MDGA2	rs3007103	$3.65x10^{-4}$
SEMA4F	rs6546920	$5.21x10^{-4}$
POU6F2	rs6946031	$6.04x10^{-4}$
LMX1B	rs1005918	$7.83x10^{-4}$
RTN4	rs7575107	$7.86x10^{-4}$
FARP2	rs740314	$8.12x10^{-4}$
ROBO2	rs6767250	$1.03x10^{-3}$
CNTN4	rs17044749	$1.08x10^{-3}$
NRP1	rs12257894	$1.38x10^{-3}$
SHH	rs2125483	$1.72x10^{-3}$
UNC5C	rs13116705	$2.59x10^{-3}$
CYFIP1	rs1009153	$4.10x10^{-3}$
YWHAG	rs13247572	$4.79x10^{-3}$
NRXN1	rs2682005	$4.87x10^{-3}$
PARD3	rs950885	$4.88x10^{-3}$
NLGN1	rs551121	$5.96x10^{-3}$
SLIT2	rs4407501	$6.59x10^{-3}$
BAIAP2	rs4075482	$7.36x10^{-3}$
CDK5	rs1549759	$7.60x10^{-3}$
LRRC4C	rs16934385	$7.63x10^{-3}$
NRTN	rs3763046	$9.09x10^{-3}$
OTX2	rs11158129	$9.33x10^{-3}$
GLI2	rs277539	$9.33x10^{-3}$
BRSK2	rs12285091	$9.66x10^{-3}$
GDNF	rs10473068	$1.08x10^{-2}$

In bold, significant angioneurins and angioneurin receptors. *NRXN3*, neurexin 3; *ATP2B2*, ATPase, Ca++ transporting, plasma membrane 2; *ROBO1*, roundabout, axon guidance receptor, homolog 1; *MDGA2*, MAM domain containing glycosylphosphatidylinositol anchor 2; *SEMA4F*, sema domain, immunoglobulin domain (Ig), transmembrane domain (TM) and short cytoplasmic domain, (semaphorin) 4F; *POU6F2*, POU class 6 homeobox 2; *LMX1B*, LIM homeobox transcription factor 1, beta; *RTN4*, reticulon 4; *FARP2*, FERM, RhoGEF and pleckstrin domain protein 2; *ROBO2*, roundabout, axon guidance receptor, homolog 2; *CNTN4*, contactin 4; *NRP1*, neuropilin 1; *SHH*, sonic hedgehog; *UNC5C*, unc-5 homolog C (C. elegans); *CYFIP1*, cytoplasmic FMR1 interacting protein 1; *YWHAG*, tyrosine 3-monooxygenase/tryptophan 5-monooxygenase activation protein, gamma; *NRXN1*, neurexin 1; *PARD3*, par-3 family cell polarity regulator; *NLGN1*, neuroligin 1; *SLIT2,* slit homolog 2 (Drosophila)*; BAIAP2,* BAI1-associated protein 2*; CDK5,* cyclin-dependent kinase 5*; LRRC4C,* leucine rich repeat containing 4C*; NRTN,* neurturin*; OTX2,* orthodenticle homeobox 2*; GLI2,* GLI family zinc finger 2*; BRSK2,* BR serine/threonine kinase 2*; GDNF,* glial cell derived neurotrophic factor.
