# Supplementary material for: Lung Transcriptomics during Protective Ventilatory Support in Sepsis-Induced Acute Lung Injury
Source: PLoS One. 2015 Jul 6;10(7):e0132296. doi: 10.1371/journal.pone.0132296 (PMC4492998; doi:10.1371/journal.pone.0132296)
Supplement: S1 Text — (DOC) [file pone.0132296.s007.doc]

**Supporting Text S1**

**Table of contents**

1. Supplementary methods

1.1 Animal preparation and experimental protocol.......................................................... 2

1.2 RNA isolation and hybridization................................................................................. 2

1.3 Differential gene expression analyses with Microarrays and Real-Time PCR validation................................................................................................................. 2

1.4 Identification of biological processes from differential gene expression analyses........................................................................................................................... 2

1.5 Inference of deregulated microRNA species in ALI and biological validation by small RNA sequencing.............................................................................................................. 3

2. Supplementary References...................................................................................................... 5

**1. Supplementary methods**

**1.1 Animal preparation and experimental protocol.** A detailed description of this experimental model is provided elsewhere [S1]. Briefly, 24 hours after CLP, surviving septic animals (n=18) had the cecum removed and were randomized to spontaneous breathing (SS, n=6), low tidal volume (6 ml/kg) plus 10 cm H_2_O PEEP (SLTV, n=6), and high tidal volume (20 ml/kg) with 2 cm H_2_O PEEP (SHVT, n=6). Sham operated animals (NA) served as non-septic controls (n=6). A cervical tracheotomy was performed using a 14-G Teflon catheter in the animals allocated to MV. Thereafter, animals were paralyzed with 1 mg/kg of pancuronium bromide and connected to a time-cycled, volume-limited rodent ventilator (Ugo Basile, Varese, Italy) and placed on a temperature controlled table to maintain body temperature at 37ºC. FiO_2_ was 0.6 in both MV groups. Ventilator rate was set at 90 cycles/min and 30 cycles/min in the SLVT and SHVT groups, respectively, to maintain constant minute ventilation and comparable PaCO_2_.

**1.2 RNA isolation and hybridization.** Total RNA was extracted using TRIreagent (Sigma-Aldrich, St. Gallen, Switzerland) following manufacturer’s instructions. Residual genomic DNA was removed by a DNase I and RNase inhibitor treatment (Amersham Biosciences, Piscataway, NJ). 75 ng of total RNA was used for cDNA synthesis using two-cycle target labeling and control reagents (Affymetrix, Santa Clara, CA) to produce biotin labeled cRNA. After quality control, 10 µg of fragmented cRNA were hybridized to the GeneChip Rat Genome 230 2.0 Array (Affymetrix, Santa Clara, CA) containing 31,000 transcript variants from 28,000 well-characterized rat genes. Hybridization was performed for 16 h at 45ºC. Each microarray was washed and stained with streptavidin-phycoerythrin in a Fluidics station 450 (Affymetrix, Santa Clara, CA) and scanned at 1.56 µm resolution in a GeneChip Scanner 3000 7G System (Affymetrix, Santa Clara, CA). Pre-processing of data was performed using GeneChip Operating Software (GCOS).

**1.3 Differential gene expression analyses with Microarrays and Real-Time PCR validation**. Complete microarray data set and experimental protocol are available in the ArrayExpress database (www.ebi.ac.uk/arrayexpress) under accession number E-MEXP-12345, according to the Minimum Information About a Microarray Experiment (MIAME) [S2].

For validation purposes, eight genes were randomly selected for real-time PCR (qPCR) reactions. β-2 microglobulin (B2m) was used as the housekeeping gene and cycle threshold (Ct) values were normalized by subtracting B2m amplification Ct to obtain the ∆Ct for each gene. Correlation between ∆Ct values obtained by qPCR and corresponding normalized intensities from the microarrays were estimated using the Spearman correlation coefficient in R.

**1.4 Identification of biological processes from differential gene expression analyses.** For modeling the protein network structure underlying the deregulated processes, a protein-protein interaction network analysis was then performed. The network similarity scores obtained from EnrichNet {Glaab, 2012 #111} were used to measure the network interconnectivity between the defined gene set and the cellular pathways mapped to the molecular interaction network. The significance threshold of this score for each experimental group was set at 1.11, 1.17 and 1.14 for SS, SLVT and SHVT, respectively, based on a linear regression of the score to the *p*-value adjusted for multiple testing assessed by FDR.

In order to validate the key biological processes found in the experimental animal model with microarray studies, common features in two independent genomic studies in critically ill patients were assessed. The first dataset included microarray results from 21 septic patients compared to data from 13 patients with sepsis-induced ALI (Gene Expression Omnibus (GEO) accession number GSE10474) [S3]. Briefly, this experiment was conducted in patients admitted to the ICU, who were intubated and receiving MV, resembling the experimental conditions in the animal model. Whole blood was obtained from each patient within 48 h of admission, and RNA was extracted for gene expression profiling. Raw data was downloaded and processed using the *affy* package with the RMA algorithm, followed by the use of GSEA (the metric 'Diff_of_classes' was used for ranking genes after 10^4^ permutations of the gene sets. Gene sets were obtained from the collection 2 'C2': Curated gene sets from Reactome) for group comparisons (septic patients vs. sepsis-induced ALI patients).

The second dataset included the summary data from the only GWAS published to date aimed to find susceptibility alleles for ALI development in humans [S4]. This GWAS was conducted using a multi-stage design, including a discovery phase with 600 trauma-induced ALI and 2266 unrelated population-based controls. For the purpose of this study, the summary association results from the genetic variants showing nominal significance at *p*≤0.01 in the discovery phase were utilized. The tool i-GSEA4GWAS [S5], which performs an optimal form of GSEA for GWAS summary data, was used to assess the correlation between pathways/gene sets and the genetic variants, considering 500 kb flanking each gene. This analysis was conducted for canonical pathways, Gene Ontology (GO) biological process, GO molecular function, and GO cellular component.

**1.5 Inference of deregulated microRNA species in ALI and biological validation by small RNA sequencing.** GSEA was used for inferring deregulated miRNA binding motifs, as defined in the Molecular Signatures Database (MSigDB), from the differentially expressed gene lists obtained from the experimental animal model. The metric 'Diff_of_classes' was used for ranking genes after 10^4^ permutations of the gene sets. Gene sets were obtained from the collection 3 'C3': Motif gene sets from 'MIR: microRNA targets'.

For small RNA sequencing, total RNA from NA and SHVT samples used for microarray studies were used. Enrichment of the small RNA fraction and the construction of sequencing libraries were performed automatically in the AB Library Builder System by using the specific Ion Total RNA-Seq Kit (Life Technologies). Integrity and concentration of total RNA and enriched small RNA was assessed in the Qubit 2.0 fluorimeter (Qubit RNA assay kit, Life Technologies) and the Bioanalyzer system (RNA 6000 Pico Kit, Agilent, Palo Alto, CA). All samples utilized had an RNA integrity number >8.5. Half volume (10 µl) of the single-stranded cDNA obtained from the AB Library Builder System was amplified for 16 cycles with the Platinum PCR SuperMix High Fidelity (Life Technologies) in a Veriti thermocycler (Life Technologies). Each library was then purified, quantified and diluted to a final concentration of 17 pM. Amplification of each sample by emulsion PCR and enrichment of the templated-ISPs fraction were applied in the OneTouch 2 and Ion OneTouch ES systems, respectively (Ion PGM Template OT2 200 Kit). Sequencing was performed in the Ion Torrent Personal Genome Machine platform (PGM) using 316 (v2) semiconductor chips (Life Technologies) imposing a total of 180 flows for each run. Post-sequencing base calling was performed on the Torrent Suite v4.0.2 (Life Technologies) yielding a >1.2 million raw reads per sample. The Partek Flow package (Partek Inc.) was used to perform adapter trimming, 3' base trimming to filter out bases with a Phred<20, and selection of reads in the size range between 16 and 30 nucleotides (to enrich for mature miRNAs). Bowtie [S6] was employed to align the reads to the *rn5* reference rat genome. The aligned reads were mapped to both the precursor- and the mature miRNA miRBase version 21. Partek Genomic Suite v6.6 software (Partek Inc.) was utilized to filter out regions represented by a small number of reads (i.e. <5 reads per kilobase of transcript per million mapped reads (RPKM), to normalize among samples for the total number of reads, and to assess differential miRNA expression using ANOVA.

**2. Supplementary References**

S1. Herrera MT, Toledo C, Valladares F, Muros M, Diaz-Flores L, Flores C, et al. Positive end-expiratory pressure modulates local and systemic inflammatory responses in a sepsis-induced lung injury model. Intensive Care Med. 2003; 29: 1345-1353.

S2. Brazma A, Hingamp P, Quackenbush J, Sherlock G, Spellman P, Stoeckert C, et al. Minimum information about a microarray experiment (MIAME)-toward standards for microarray data. Nat Genet. 2001; 29: 365-371.

S3. Howrylak JA, Dolinay T, Lucht L, Wang Z, Christiani DC, Sethi JM, et al. Discovery of the gene signature for acute lung injury in patients with sepsis. Physiol Genomics. 2009; 37: 133-139.

S4. Christie JD, Wurfel MM, Feng R, O'Keefe GE, Bradfield J, Ware LB, et al. Genome wide association identifies PPFIA1 as a candidate gene for acute lung injury risk following major trauma. PLoS One. 2012; 7: e28268.

S5. Zhang K, Cui S, Chang S, Zhang L, Wang J. i-GSEA4GWAS: a web server for identification of pathways/gene sets associated with traits by applying an improved gene set enrichment analysis to genome-wide association study. Nucleic Acids Res. 2010; 38: W90-95.

S6. Langmead B, Trapnell C, Pop M, Salzberg SL. Ultrafast and memory-efficient alignment of short DNA sequences to the human genome. Genome Biol. 2009; 10: R25.
